# Early Postoperative Serum Carcinoembryonic Antigen Is a Stronger Independent Prognostic Factor for Stage II Colorectal Cancer Patients Than T4 Stage and Preoperative CEA

**DOI:** 10.3389/fonc.2021.758509

**Published:** 2022-01-11

**Authors:** Du Fenqi, Liu Yupeng, Zhang Qiuju, Yuan Chao, Song Wenjie, Xia Tianyi, Guo Junnan, Xue Weinan, Jiang Xiufeng, Bai Junge, Jia Chenyang, Xi Hua, Li Yien, Bai Xuefeng, Liu Yanlong

**Affiliations:** ^1^ Department of Colorectal Surgery, Harbin Medical University Cancer Hospital, Harbin, China; ^2^ Department of Epidemiology and Biostatistics, School of Public Health and Management, Wenzhou Medical University, Wenzhou, China; ^3^ Department of Biostatistics, Public Health School of Harbin Medical University, Harbin, China; ^4^ Department of Anus and Intestine Surgery, The First Hospital of Qiqihar, Qiqihar, China; ^5^ Department of Epidemiology, Public Health School of Harbin Medical University, Harbin, China

**Keywords:** Postoperative serum carcinoembryonic antigen, stage II colorectal cancer, prognosis, high-risk factor, adjuvant chemotherapy

## Abstract

**Background:**

Serum carcinoembryonic antigen (CEA) is an important biomarker for diagnosis, prognosis, recurrence, metastasis monitoring, and the evaluation of the effect of chemotherapy in colorectal cancer (CRC). However, few studies have focused on the role of early postoperative CEA in the prognosis of stage II CRC.

**Methods:**

Patients with stage II CRC diagnosed between January 2007 and December 2015 were included. Receiver operating characteristic (ROC) curves were used to obtain the cutoff value of early postoperative CEA, CEA ratio and CEA absolute value. The areas under curves (AUCs) were used to estimate the predictive abilities of the CEA and T stage. The stepwise regression method was used to screen the factors included in the Cox regression analysis. Before and after propensity score (PS) - adjusted Cox regression and sensitivity analysis were used to identify the relationship between early postoperative CEA and prognosis. Meta-analysis was performed to verify the results. Kaplan-Meier survival curves were used to estimate the effects of CEA on prognosis.

**Results:**

We included 1081 eligible patients. ROC curves suggested that the cutoff value of early postoperative CEA was 3.66 ng/ml (P <0.001) and the AUC showed early postoperative CEA was the most significant prognostic marker in stage II CRC (P = 0.0189). The Cox regression and sensitivity analysis before and after adjusting for PS both revealed elevated early postoperative CEA was the strongest independent prognostic factor of OS, DFS, and CSS (P < 0.001). Survival analysis revealed that patients with elevated early postoperative CEA had lower OS (53.62% VS 84.16%), DFS (50.03% VS 86.75%), and CSS (61.77% VS 90.30%) than patients with normal early postoperative CEA (P < 0.001). When the postoperative CEA was positive, the preoperative CEA level showed no significant effect on the patient’s prognosis (all P-values were > 0.05). Patients with a CEA ratio ≤0.55 or CEA absolute value ≤-0.98 had a worse prognosis (all P-values were < 0.001). Survival analysis suggested that adjuvant chemotherapy for stage II CRC patients with elevated early postoperative CEA may improve the CSS (P = 0.040).

**Conclusions:**

Early postoperative CEA was a better biomarker for prognosis of stage II CRC patients than T stage and preoperative CEA, and has the potential to become a high-risk factor to guide the prognosis and treatment of stage II CRC patients.

## Introduction

As the third most common malignant tumor in the world, colorectal cancer (CRC) poses a serious threat to human health due to its high morbidity and mortality (1). Radical resection is the primary treatment for non-metastatic CRC. Adjuvant chemotherapy is proven to show higher survival benefits in stage III patients, and the survival of stage II colon cancer patients with high-risk factors (HRFs) may be improved through adjuvant chemotherapy (2, 3). Due to the existence of HRFs, the prognosis of patients with stage II CRC is heterogeneous. Therefore, it is necessary to clarify which factors can be defined as HRFs for the guidance of the prognosis and treatment for stage II CRC.

Carcinoembryonic antigen (CEA) is a tumor-associated antigen, which was first extracted from colon cancer and embryonic tissues by Gold and Freedman in 1965. It is related to the progression of various solid tumors (4). Serum CEA is an important biomarker for diagnosis, prognosis, recurrence, metastasis monitoring, and the evaluation of the effect of chemotherapy in CRC (5–8). Recently, a few studies showed that postoperative CEA is an important prognostic factor for CRC (9–13). However, there are no studies evaluating the guided and predictive values of postoperative CEA on the prognosis of stage II CRC, and its potential to be a new HRF for stage II CRC patients remains unclear. Therefore, we conducted a single-center retrospective study to explore the effects of early postoperative CEA on the prognosis and its guiding value for adjuvant chemotherapy in stage II CRC patients.

## Methods

### Data Collection

The current study was a single-center retrospective clinical study, and all patients met the following conditions: (1) they received radical surgical treatment for colorectal cancer at the Harbin Medical University Cancer Hospital between January 2007 and December 2015, and they were pathologically diagnosed as stage II CRC; (2) serum CEA was tested before and within 3 months after surgery; (3) the patients had complete follow-up records as recommended by the CSCO guidelines; (4) they agreed to provide informed consent. The exclusion criteria were as follows: (1) unclear diagnosis of the pathological type; (2) CEA values not available; (3) received neoadjuvant therapy; (4) recurrence or metastasis within 3 months after surgery; (5) no follow-up information; (6) concomitant other cancers at initial diagnosis. Eventually, 1081 patients were included in this study.

The following information was obtained from the electronic medical record system (EMRS) and the telephone follow-up database of the Harbin Medical University Cancer Hospital: (1) gender, age, height, weight, smoking history, drinking history, and other individual characteristics; (2) disease information, such as concomitant diseases, tumor sites, surgery time and approaches, postoperative pathological reports, and available CEA values; (3) follow-up information. Postoperative pathological reports included T and N stages, pathological type, differentiation degree, upper and lower resection margins, lymphatic infiltration, vascular infiltration, and the number of lymph node dissections. The pathological stage was defined according to the criteria in the 8th American Joint Committee on Cancer (AJCC) manual for CRC.

### Statistical Analysis

All data in the current study were analyzed using the IBM SPSS STATISTICS 23.0 software, and MedCalc 18.2.1 was used for processing the images. The study endpoints were overall survival (OS), disease-free survival (DFS), and cancer-specific survival (CSS).

Receiver operating characteristic curve (ROC)’s area under curves (AUCs) were used to evaluate the predictive efficacy of early postoperative CEA in the prognosis of CRC. When AUC >0.9, the predictive efficacy was considered superior, when it was between 0.7 and 0.9, the predictive efficacy was considered good, and when it was between 0.5 and 0.7, the predictive efficacy is considered satisfactory (14). The optimal cutoff value of early postoperative CEA, CEA ratio, and CEA absolute values suggested by the ROC curve were 3.66 ng/ml, 0.55, and -0.98. And the Kaplan-Meier survival curves were used to estimate the survival in the different groups. A P <0.05 was considered statistically significant.

When performing Cox regression analysis, a stepwise regression method was used to obtain the final multivariate model, and the variables with P <0.05 were retained in the final model. Early postoperative CEA was considered as a primary covariate and other factors were adjusted according to the propensity score (PS). The PS score was considered as another covariate, which was included in the model along with the early postoperative CEA to construct Cox proportional hazards regression models with different outcomes. These factors included preoperative CEA, gender, age, BMI, tumor sites, histological type, differentiation degree, T stage, and adjuvant chemotherapy. Subsequently, the PS-adjusted regression results were verified using PS stratification and inverse probability weighting (IPTW).

A meta-analysis was performed using Comprehensive Meta-Analysis (CMA) 3.3.070. The keywords “postoperative serum CEA, colorectal cancer, prognosis” and “serum CEA, colorectal cancer, prognosis” were used as index words to search for target publications in PubMed. The meta-analysis was performed by statisticians.

## Results

### Patient Characteristics

**Figure 1:**
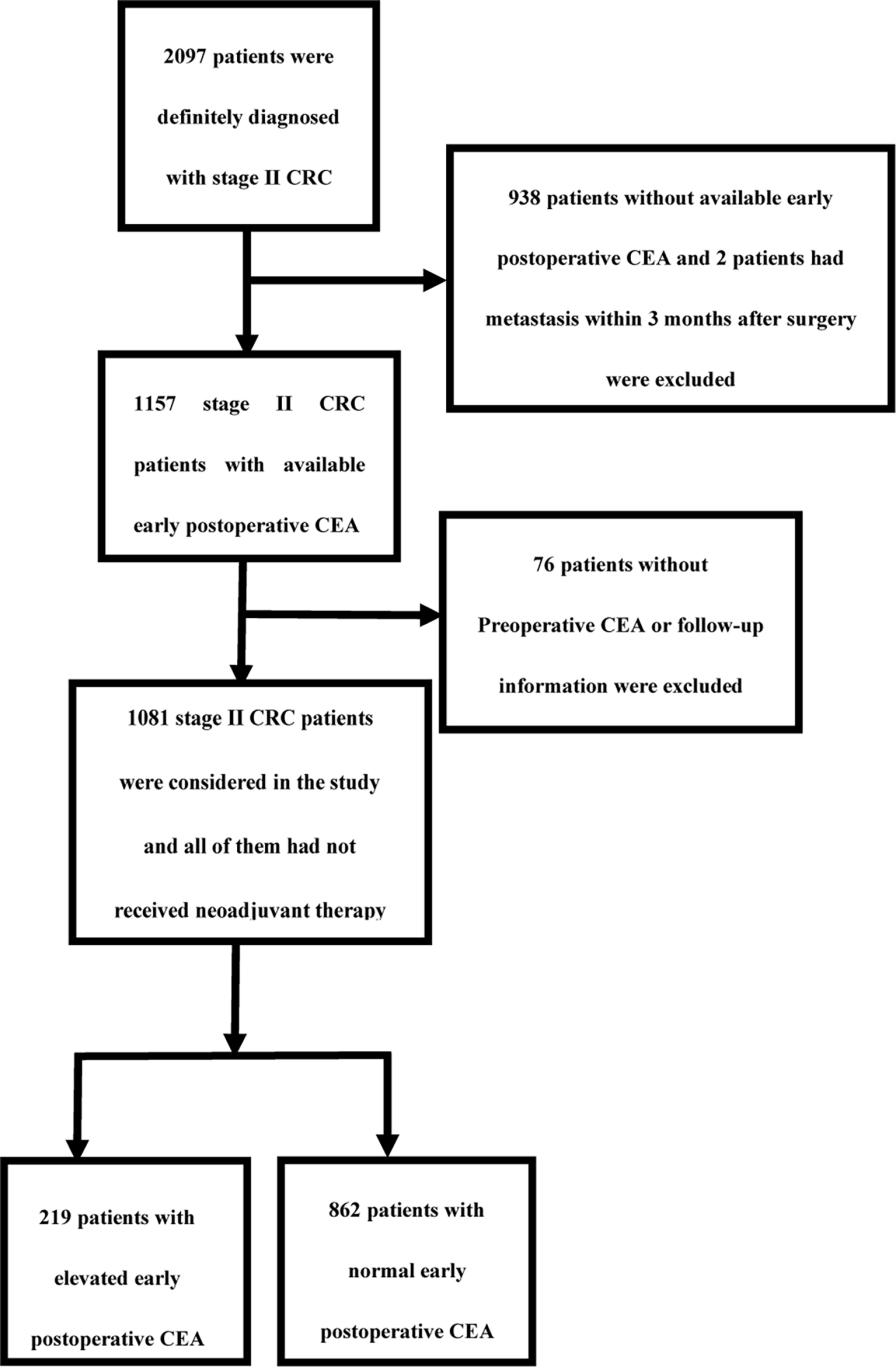
Study design.

A total of 1081 stage II CRC patients were considered in the study (**Figure 1**). The mean age of the patients was 57 y (11 y – 87 y). A total of 436 patients (40.3%) had stage T3 CRC and 645 patients (59.7%) had stage T4. An early postoperative CEA of ≤3.66 ng/ml was observed in 862 patients (79.7%), and that >3.66 ng/ml in 219 patients (20.3%). A total of 207 patients (19.1%) received 3-month chemotherapy and 475 patients (43.94%) received 6-month chemotherapy. During the final follow-up, it was found that 235 patients (21.74%) showed recurrence and metastasis. A total of 228 (21.09%) patients eventually died of neoplastic causes and 71 patients (6.57%) died of non-neoplastic causes. The median follow-up time was 2125 days. The 5-year OS was 78.11%, the 5-year DFS was 79.65%, and the 5-year CSS was 84.80%. The demographic and clinical characteristics of the patients are summarized in **Table 1**.

**Table 1:** Patient demographic and clinicopathologic data.

Variables	Patients (N = 1,081)
**Gender n (%)**	
Male	671 (62.07)
Female	410 (37.93)
**Age n (%)**	
>60	632 (58.46)
≤60	449 (41.54)
**BMI (kg/m^2^) n (%)**	
<18.5	53 (4.90)
18.5-23.9	572 (52.91)
24-27.9	343 (31.73)
≥28	113 (10.46)
**Location n (%)**	
Right colon	328 (30.34)
Left colon	330 (30.53)
Rectum	423 (39.13)
**T stage n (%)**	
T3	437 (40.43)
T4	644 (59.57)
**Pathological type n (%)**	
Mucinous adenocarcinoma and signet ring cell carcinoma	262 (24.23)
Adenocarcinoma	819 (75.77)
**Differentiation n (%)**	
Poor	136 (12.58)
Moderate	906 (83.81)
Well	39 (3.61)
**Preoperative CEA level n (%)**	
>5 ng/ml	409 (62)
≤ 5 ng/ml	672 (38)
**Postoperative CEA level n (%)**	
>3.66 ng/ml	864 (79.93)
≤3.66 ng/ml	217 (20.07)
**Adjuvant chemotherapy n (%)**	
No adjuvant chemotherapy	399 (36.91)
Three-month adjuvant chemotherapy	207 (19.15)
Six-month adjuvant chemotherapy	475 (43.94)
**Metastasis or recurrence n (%)**	
Yes	224 (20.72)
No	857 (79.28)
**Survival status n (%)**	
Alive	784 (72.53)
Dead	297 (27.47)

### ROC Curves Suggested That Early Postoperative CEA Is a More Significant Predictor of Prognosis of Stage II CRC Than T Stage and Preoperative CEA

**Figure 2:**
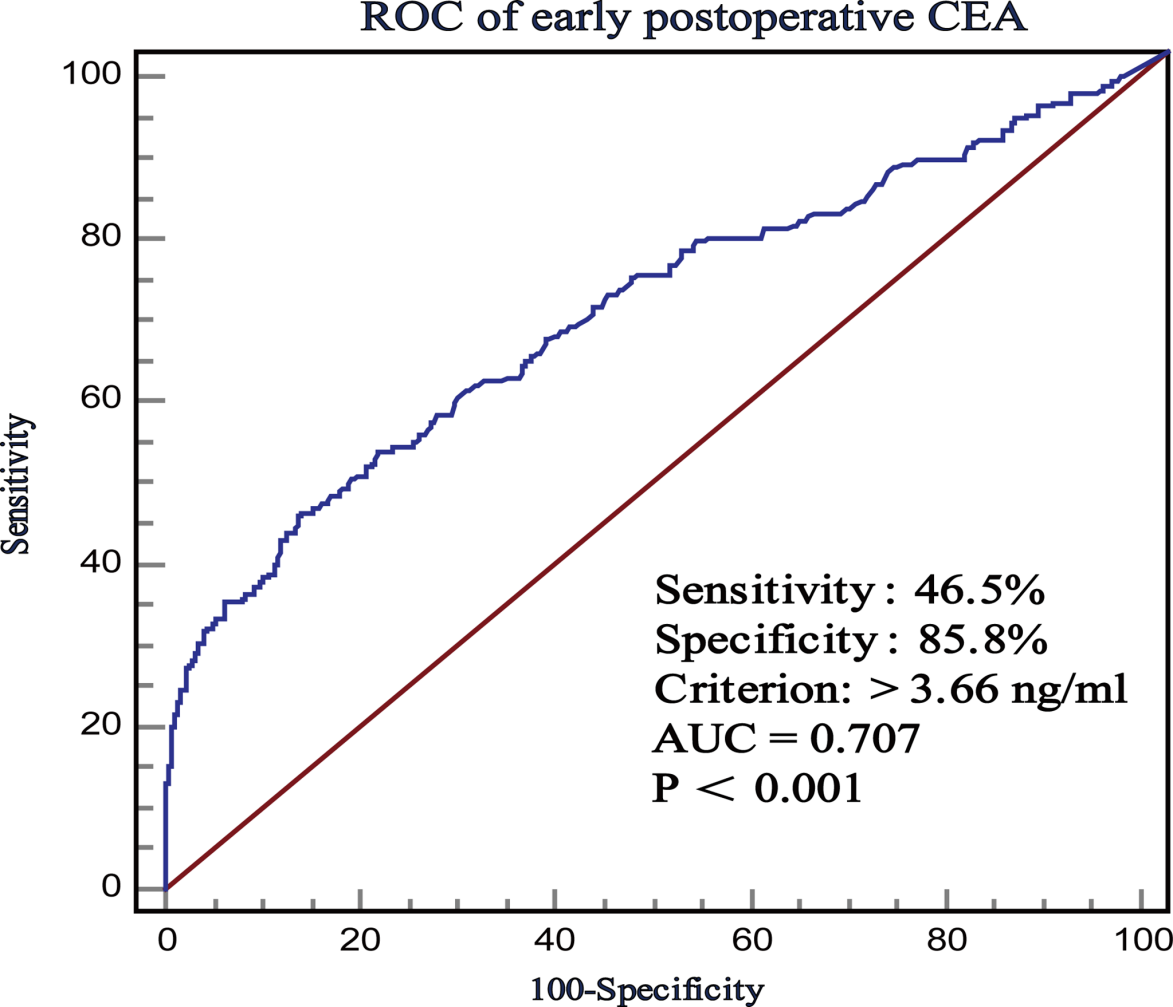
The receiver operating characteristics (ROC) curves of postoperative CEA with respect to CSS.

**Figure 3:**
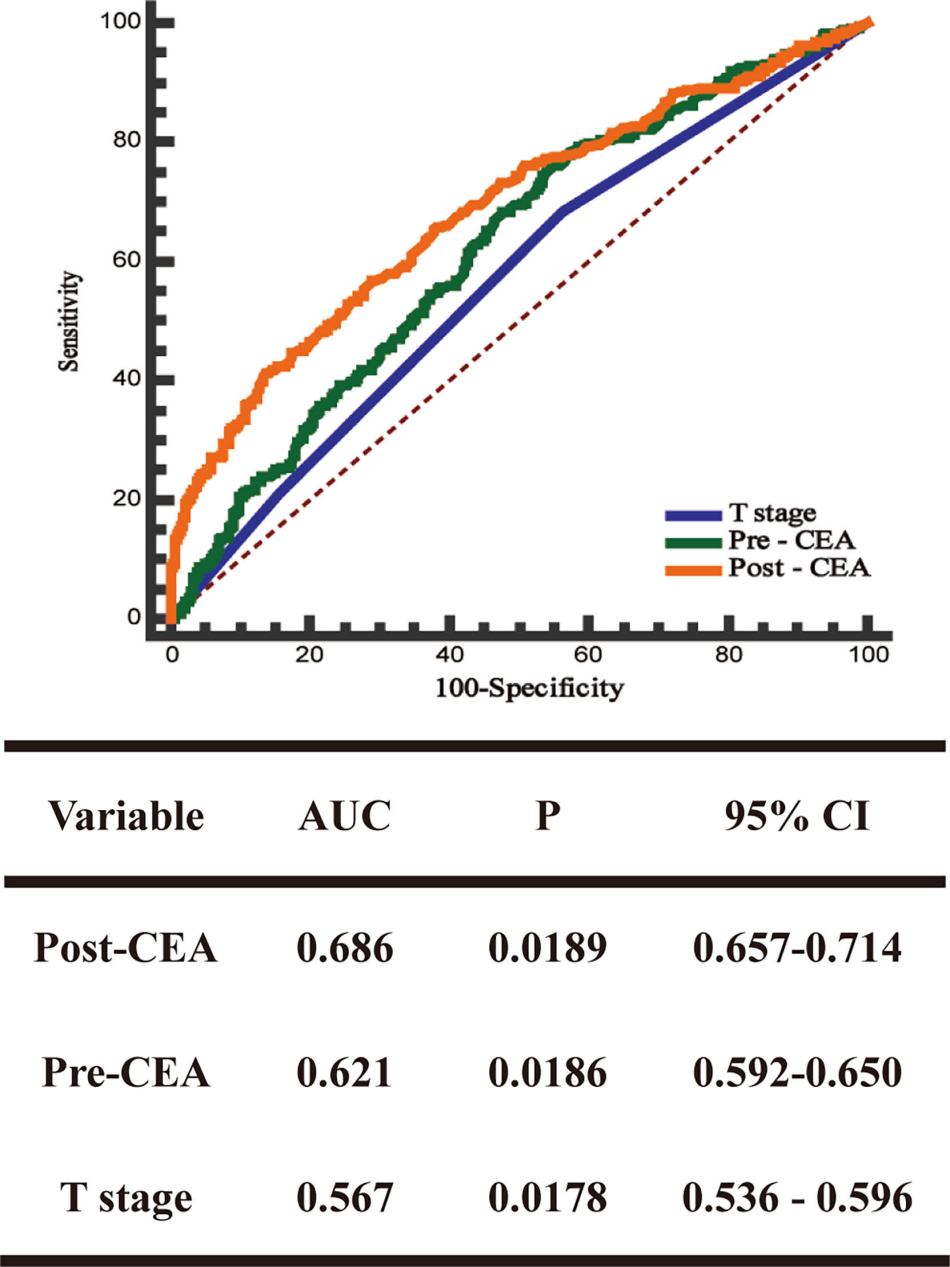
The receiver operating characteristics (ROC) curves and the area under the curves (AUCs) of postoperative CEA, preoperative CEA, and T stage.

ROC curve was used to evaluate the predictive effects of early postoperative CEA, T stage, and preoperative CEA on the prognosis of stage II CRC. In the case of CSS, according to the ROC curve, the best cutoff value of early postoperative CEA was 3.66 ng/ml, with a sensitivity and specificity of 46.5% and 85.84%, respectively (**Figure 2**). Subsequently, we used the ROC curves to compare the predictive effects of early postoperative CEA, T stage, and preoperative CEA. AUCs showed that the early postoperative CEA better predicted the prognosis of stage II CRC (AUC > 0.686; 95% CI, 0.657-0.714) and it was significantly better than that using T stage (AUC > 0.621; 95% CI, 0, 592-0.650) and preoperative CEA (AUC > 0.686; 95% CI, 0.657 - 0.714) (**Figure 3**).

### Univariate and Multivariate Analyses Revealed That Elevated Early Postoperative CEA Was the Most Significant Independent Prognostic Factor for Stage II CRC Patients

To identify the relationships between early postoperative CEA and prognosis in stage II CRC, we performed univariate and multivariate Cox regression analyses before and after adjusting for PS, followed by sensitivity analysis. The results after adjusting for PS were verified using PS stratification and IPTW.

Before adjusting for PS, univariate Cox regression analyses results showed that age, tumor sites, histological type, differentiation degree, T4 stage, adjuvant chemotherapy, elevated early postoperative CEA, and elevated preoperative CEA were prognostic factors for OS, DFS, and CSS (all P-values were < 0.05, **Table 2**). Multivariable Cox regression analysis results indicated that elevated early postoperative CEA was the most significant independent prognostic factor for OS (HR = 2.59; 95% CI, 2.006-3.343, P = 0.000), more significant than the T4 stage and elevated preoperative CEA. Sensitivity analysis showed that the standardized regression coefficient of early postoperative CEA was 0.068, which was significantly greater than that of the other factors, supporting the conclusion that early postoperative CEA is the most significant independent prognostic factor for OS. Similarly, elevated early postoperative CEA was also the most significant independent prognostic factor for DFS (HR = 4.505; 95% CI, 3.375-6.015, P = 0.000, standardized regression coefficient = 0.122) and CSS (HR = 3.943; 95% CI, 2.901-5.357, P = 0.000, sensitivity standardized regression coefficient = 0.118) (**Table 3**).

**Table 2:** Univariate Cox regression analysis before PS for overall survival (OS), disease-free survival (DFS) and cancer specific survival (CSS).

Factors	OS		DFS		CSS	
	P-value	HR (95%CI)	P-value	HR (95%CI)	P-value	HR (95%CI)
**Gender**	0.164	1.183 (0.934-1.500)	0.096	1.264 (0.959-1.666)	0.201	1.209 (0.904-1.616)
**Age**	1.941	2.015 (10603-2.532)	0.010	1.412 (1.087-1.834)	0.000	1.747 (1.322-2.307)
**BMI**						
<18.5	0.136	1.424 (0.894-2.268)	0.339	1.323 (0.745-2.349)	0.342	1.337 (0.735-2.433)
18.5-23.9 (ref.)						
24-27.9	0.468	0.909 (0.703-1.176)	0.509	1.103 (0.825-1.475)	0.986	1.003 (0.734-1.370)
≥28	0.733	0.933 (0.628-1.387)	0.731	1.080 (0.695-1.678)	0.662	1.108 (0.699-1.758)
**Smoking**	0.212	1.166 (0.916-1.485)	0.247	1.178 (0.893-1.554)	0.343	1.154 (0.858-1.552)
**Alcohol**	0.780	1.040 (0.791-1.367)	0.543	1.102 (0.806-1.505)	0.595	1.093 (0.787-1.520)
**Hypertension history**	0.132	1.271 (0.930-1.736)	0.243	1.236 (0.866-1.766)	0.165	1.308 (0.896-1.909)
**Diabetes history**	0.131	1.350 (0.914-1.992)	0.293	1.279 (0.808-2.025)	0.189	1.373 (0.856-2.203)
**Site (0-colon,1-rectum)**	0.000	1.547 (1.232-1.943)	0.005	1.452 (1.118-1.886)	0.000	1.652 (1.251-2.182)
**Diameter (cm)**	0.572	1.069 (0.847-1.349)	0.465	1.105 (0.846-1.442)	0.597	1.080 (0.813-1.435)
**Histomorphology**	0.000	1.523 (1.206-1.924)	0.010	1.429 (1.091-1.874)	0.002	1.555 (1.169-2.067)
**Differentiation**	0.000	1.725 (1.328-2.240)	0.001	1.677 (1.233-2.282)	0.002	1.672 (1.211-2.308)
**Lymphadenectasis**	0.997	1.000 (0.751-1.333)	0.253	1.200 (0.878-1.639)	0.553	1.109 (0.789-1.559)
**T stage**						
T4a	0.031	1.334 (1.027-1.732)	0.006	1.527 (1.129-2.066)	0.011	1.524 (1.102-2.107)
T4b	0.005	1.577 (1.145-2.170)	0.002	1.764 (1.223-2.544)	0.008	1.712 (1.150-2.107)
**Adjuvant chemotherapy**	0.000	0.627 (0.498-0.789)	0.041	0.759 (0.582-0.989)	0.006	0.674 (0.508-0.895)
**Preoperative CEA**	0.000	1.794 (1.430-2.252)	0.000	1.697 (1.307-2.204)	0.000	1.746 (1.323-2.305)
**Early postoperative CEA**	0.000	3.569 (2.826-4.508)	0.000	5.084 (3.910-6.609)	0.000	4.950 (3.741-6.549)

**Table 3:** Multivariate Cox regression analysis before PS and sensitivity analyses for overall survival (OS), disease-free survival (DFS) and cancer specific survival (CSS).

Factors	OS		Sensitivity Analysis	DFS		Sensitivity Analysis	CSS		Sensitivity Analysis
	P-value	HR (95%CI)	Standardized regression coefficient	P-value	HR (95%CI)	Standardized regression coefficient	P-value	HR (95%CI)	Standardized regression coefficient
**Age**	0.000	1.949(1.532-2.480)	0.045	0.010	1.412(1.087-1.834)	0.022	0.000	1.747(1.250-2.250)	0.043
**Site(0-colon,1-rectum)**	0.000	1.670(1.306-2.135)	0.035	0.005	1.452(1.118-1.886)	0.033	0.000	1.652(1.269-2.306)	0.045
**Histomorphology**	0.002	1.448(1.140-1.839)	0.025	0.010	1.429(1.091-1.874)	0.020	0.002	1.555(1.059-1.898)	0.029
**Differentiation**	0.000	1.856(1.408-2.448)	0.048	0.001	1.677(1.233-2.282)	0.041	0.002	1.672(1.207-2.389)	0.051
**T4a**	0.127	1.237(0.942-1.624)	0.016	0.006	1.527(1.129-2.066)	0.030	0.011	1.524(1.000-1.958)	0.032
**T4b**	0.015	1.534(1.086-2.166)	0.042	0.002	1.764(1.223-2.544)	0.065	0.008	1.712(1.143-2.674)	0.067
**Adjuvant chemotherapy**	0.070	0.792(0.616-1.019)	0.016	0.041	0.759(0.582-0.989)	0.000	0.006	0.674(0.656-1.220)	0.010
**CEA before-surgery**	0.009	1.376(1.083-1.750)	0.022	0.000	1.697(1.307-2.204)	0.004	0.000	1.746(0.885-1.593)	0.014
**CEA post-surgery**	0.000	2.590(2.006-3.343)	0.068	0.000	5.084(3.910-6.609)	0.122	0.000	4.950(2.901-5.357)	0.118

Subsequently, according to the cutoff value of early postoperative CEA, patients were classified into normal and elevated groups. After other factors were adjusted using the propensity score (PS), the PS score was considered as another covariate, which was included in the regression analysis along with early postoperative CEA. The results showed that after adjusting for PS, early postoperative CEA was still an independent prognostic factor for OS (HR = 2.830; 95% CI, 2.202-3.637, P = 0.000), DFS (HR = 4.552, 95% CI, 3.427-6.046, P = 0.000), and CSS (HR = 4.186; 95% CI, 3.102-5.648, P = 0.000). The same results were obtained after verification using PS stratification and IPTW (**Table 4**).

**Table 4:** Cox regression analysis after PS-adjusted.

Outcome	PS-adjusted regression	Sensitivity analyses by other PS-based methods	
postoperative CEA	PS-adjusted regression HR (95% CI), P-value	PS-stratified HR (95% CI), P-value	IPTW- HR (95% CI), P-value
CSS	4.186 (3.102-5.648), 0.000	4.044 (3.000-5.452), 0.000	3.112 (2.337-4.142), 0.000
DFS	4.552 (3,427-6.046), 0.000	4.518 (3.411-5.984), 0.000	3.333 (2.540-4.373), 0.000
OS	2.830 (2.202-3.637), 0.000	2.818 (2.198-3.612), 0.000	2.526 (2.008-3.178), 0.000

We used propensity score (PS)-adjusted regression adjusted preoperative CEA, gender, age, BMI, tumor sites, histological type, differentiation degree, T stage and chemotherapy and got a PS score, which was considered as another covariate and included in the model along with the early postoperative CEA to construct Cox proportional hazards regression models with different outcomes. The PS-adjusted regression results were verified using PS stratification and inverse probability weighting (IPTW).

### Meta-Analysis Verified That Postoperative CEA Was an Independent Prognostic Factor for CRC

**Figure 4:**
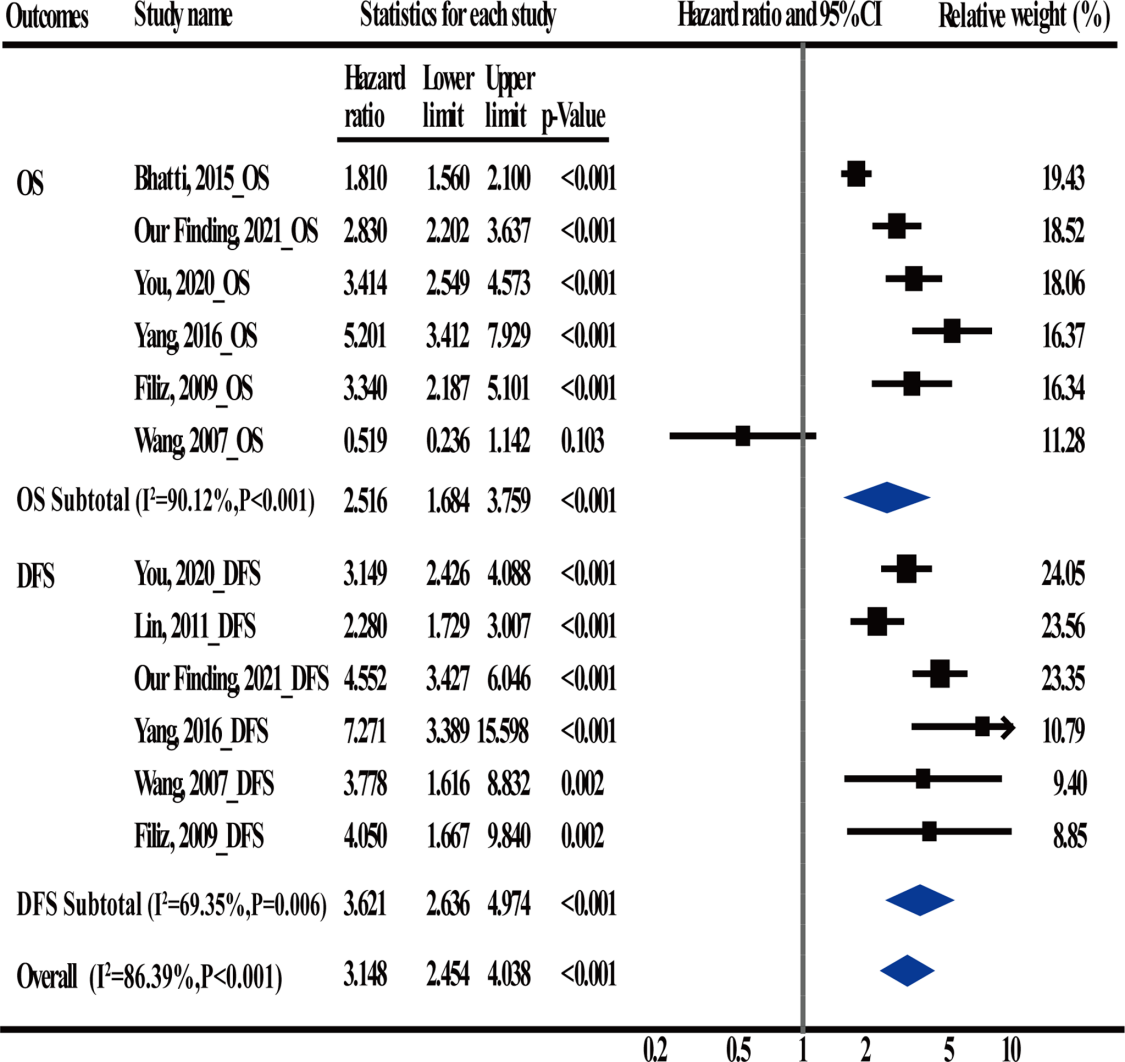
Meta-Analysis estimate postoperative carcinoembryonic antigen (CEA) levels serves as an independent prognostic factor in CRC patients.

A total of 5 articles were included in the meta-analysis to verify the conclusions obtained using the univariate and multivariable Cox regression analyses. Information regarding these 5 articles is presented in **Table 5**. In the case of OS, the heterogeneity test results showed that there were significant differences among studies (I^2^ = 90.12%; P < 0.001); therefore, the random-effects model was adopted. The HR (2.516) and 95% CI (1.684, 3.759) (P < 0.001) suggested that postoperative CEA was an independent prognostic factor for OS. In the case of DFS, the heterogeneity test results showed that there were significant differences among studies (I^2^ = 69.35%; P = 0.006); therefore, the random-effects model was adopted. The HR (3.621) and 95% CI (2.636, 4.974) (P < 0.001) suggested that postoperative CEA was an independent prognostic factor for DFS (**Figure 4**).

**Table 5:** Baseline characteristics of meta-analysis articles.

Study author and publication year	Country/Region	Study design	Study period	Sample size	Gender(Male/Female)	postoperative CEA’ scut-off value	Multivariable analysis results of postoperative CEA-OSHR (95% CI)	Multivariable analysisresults of postoperative CEA-DFSHR (95% CI)
Bhatti 2015	UK	retrospective study	2008.12 - 2011.12	569	289/280	5ng/ml (≤5-ref.)	1.810 (1.650-2.120)	–
You 2020	China	retrospective study	2009.1-2015.12	1008	605/403	5ng/ml (≤5-ref.)	3.414 (2.549-4.574)	3.149 (2.426-4.088)
Yang 2016	Korea	retrospective study	1999.1-2008.12	318	189/129	6ng/mL (≤6-ref.)	5.201 (3.412–7.929)	7.271 (3.389-15.597)
Filiz 2009	Turkey	retrospective study	2002.2-2006.6	114	70/44	5ng/ml (<5-ref.)	3.340 (2.187-5.101)	4.050 (1.667-9.841)
Lin 2011	Taiwan	prospective study	2000-2004	1361	897/464	5ng/mL (≤5-ref)	–	2.280 (1.730-3.010)
Wang 2007	Taiwan	retrospective study	2001.1-2006.6	425	210/215	5ng/mL (<5-ref.)	0.519 (0.236–1.143)	3.778 (1.616-8.831)

### Survival Analysis Revealed That the Patients With Elevated Early Postoperative CEA Had a Worse Prognosis

**Figure 5:**
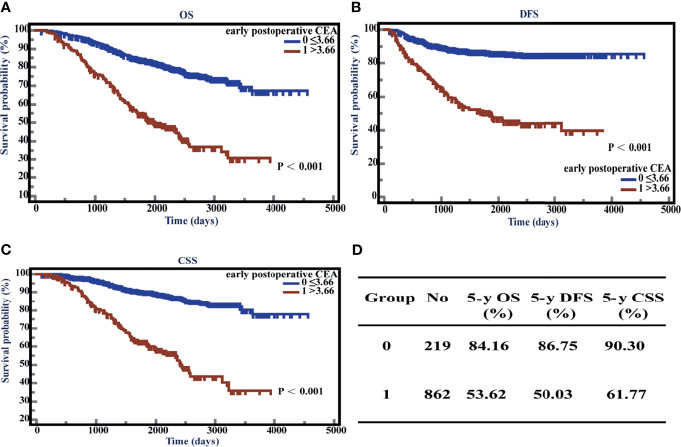
Overall survival (OS), disease-free survival time (DFS), and cancer-specific survival (CSS) with respect to postoperative carcinoembryonic antigen (CEA) levels. **(A)** K-M curves of OS based on postoperative CEA levels. **(B)** K-M curves of DFS based on postoperative CEA levels. **(C)** K-M curves of CSS based on postoperative CEA levels. **(D)** 5-year OS, DFS, and CSS of normal and elevated groups.

To assess the effects of elevated early postoperative CEA on the survival time in stage II CRC patients, we performed Kaplan-Meier (K-M) survival analysis using the follow-up information. The patients showed poor prognosis when early postoperative CEA was > 3.66 ng/ml. The 5-year OS, DFS, and CSS of the patients with elevated early postoperative CEA were 53.62%, 50.03%, and 61.77%, respectively, which were significantly lower than those of patients with normal early postoperative CEA (the 5-year OS, DFS, and CSS were 84.16%, 86.75%, and 90.30%, respectively). All P-values were < 0.001 (**Figures 5A–D**).

Subsequently, we conducted further analysis on 219 patients with positive early postoperative CEA. Among these patients, 140 patients had a positive preoperative CEA and 79 had a negative preoperative CEA. Kaplan-Meier (K-M) survival analysis showed that 5-year OS, DFS, and CSS of patients with positive early postoperative CEA and positive preoperative CEA were 48.90%, 51.23%, and 59.18%, respectively, and of patients with positive early postoperative CEA and negative preoperative CEA patients were 61.54%, 47.80%, and 66.02%, respectively. K-M curves showed that there was no significant difference between the two subgroups (All P-values were >0.05 and the K-M curves are shown in **Supplementary Figure 1**).

### Patients With CEA Ratio ≤0.55 or CEA Absolute Value ≤-0.98 Had a Worse Prognosis

We further studied the survival conditions of patients who had different CEA ratio (preoperative serum CEA divided by early postoperative serum CEA) and CEA absolute value (early postoperative serum CEA subtracted from preoperative serum CEA). We used ROC curves to find the optimal cutoff value of the CEA ratio and CEA absolute value. When the event was OS, according to the ROC curve, the optimal cutoff value of CEA ratio and CEA absolute values were 0.55 and -0.98, with the sensitivity of 22.56% and 24.92%, respectively, and the specificity of 95.15% and 95.15%, respectively (**Supplementary Figure 2**). When 0.55 was used as the optimal cutoff value of CEA ratio, Kaplan-Meier (K-M) survival analysis showed that the 5-year OS, DFS, and CSS of the patients with CEA ratio ≤0.55 were 50.30%, 35.83%, and 51.84%, respectively, and of patients with CEA ratio >0.55 were 81.28%, 84.62%, and 88.68%, respectively. When -0.98 was used as the optimal cutoff value of CEA absolute value, the 5-year OS, DFS, and CSS of the patients with CEA absolute value ≤0.98 were 47.58%, 33.11%, and 50.39%, respectively, and of patients with CEA absolute value >-0.98 were 81.79%, 85.10%, and 88.98%, respectively. K-M curves illustrated that patients with CEA ratio ≤0.55 or CEA absolute value ≤-0.98 had a worse OS, DFS, and CSS than CEA ratio >0.55 or CEA absolute value >-0.98 (p < 0.001) (**Supplementary Figure 3**).

### Adjuvant Chemotherapy for Stage II CRC Patients With Elevated Early Postoperative CEA Improved CSS

**Figure 6:**
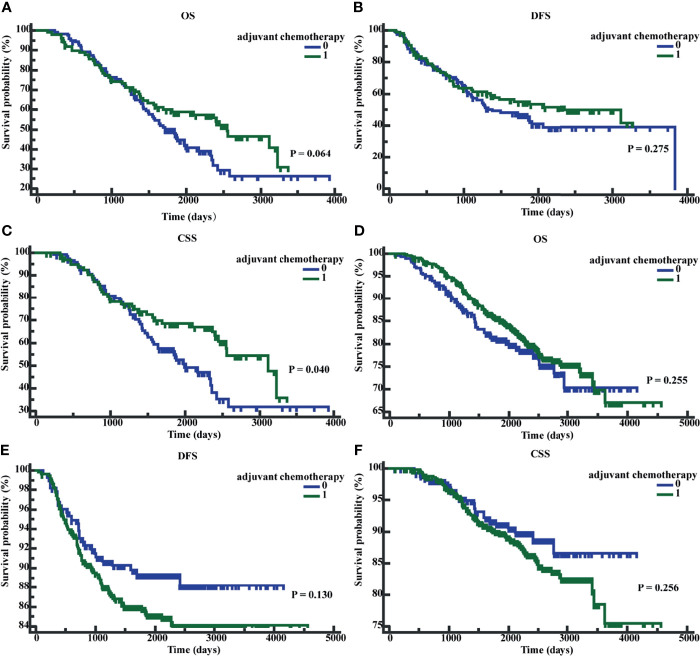
Patients’ overall survival (OS), disease-free survival time (DFS), and cancer-specific survival (CSS) based on whether receive adjuvant chemotherapy. **(A)** K-M curves of OS based on whether receive adjuvant chemotherapy in postoperative CEA-positive patients. **(B)** K-M curves of DFS based on whether receive adjuvant chemotherapy in postoperative CEA -positive patients. **(C)** K-M curves of CSS based on whether receive adjuvant chemotherapy in postoperative CEA -positive patients. **(D)** K-M curves of OS based on whether receive adjuvant chemotherapy in postoperative CEA -negative patients. **(E)** K-M curves of DFS based on whether receive adjuvant chemotherapy in postoperative CEA -negative patients. **(F)** K-M curves of CSS based on whether receive adjuvant chemotherapy in postoperative CEA -negative patients. 0, without adjuvant chemotherapy; 1, with adjuvant chemotherapy.

**Figure 7:**
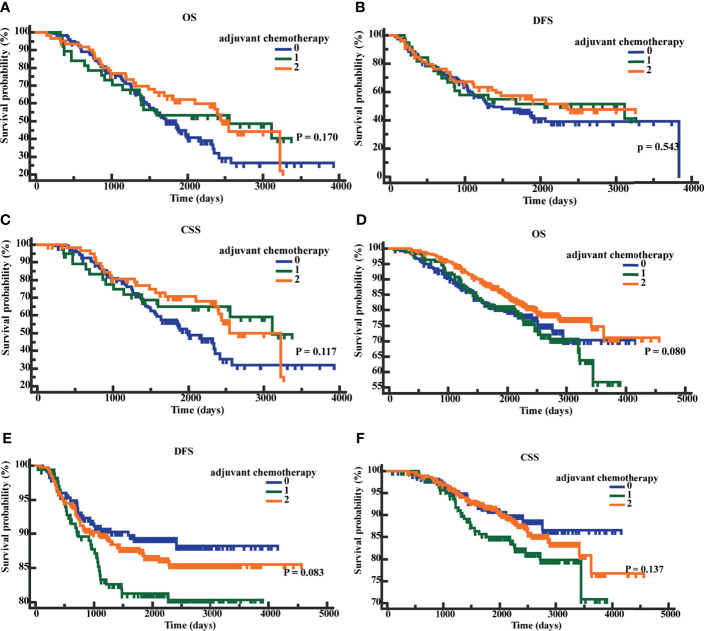
Patients’ overall survival (OS), disease-free survival time (DFS), and cancer-specific survival (CSS) based on different chemotherapy regimens. **(A)** K-M curves of OS based on different chemotherapy regimens in postoperative CEA-positive patients. **(B)** K-M curves of DFS based on different chemotherapy regimens in postoperative CEA-positive patients. **(C)** K-M curves of CSS based on different chemotherapy regimens in postoperative CEA-positive patients. **(D)** K-M curves of OS based on different chemotherapy regimens in postoperative CEA-negative patients. **(E)** K-M curves of DFS based on different chemotherapy regimens in postoperative CEA -negative patients. **(F)** K-M curves of CSS based on different chemotherapy regimens in postoperative CEA -negative patients. 0, without adjuvant chemotherapy; 1, with 3-month adjuvant chemotherapy; 2, with 6-month adjuvant chemotherapy.

We performed K-M survival analyses to explore the significance of early postoperative CEA on adjuvant chemotherapy. The K-M analysis suggested that in patients with early postoperative CEA >3.66 ng/ml, compared to patients without adjuvant chemotherapy, patients with adjuvant chemotherapy showed an improved CSS (P = 0.040; HR = 0.6501; 95% CI, 0.4339 - 0.9741). However, the differences in OS (P = 0.0623; HR = 0.7081; 95% CI, 0.4935-1.0160) and DFS (P = 0.2745; HR = 0.8107, 95%CI, 0.5572 -1.1795) were not significantly different (**Figures 6A–C**). In patients with early postoperative CEA ≤3.66 ng/ml, the differences in OS (P = 0.2543; HR = 0.8345; 95% CI, 0.6055-1.1526), DFS(P = 0.1280, HR = 1.3836, 95% CI, 0.9353-2.0467), and CSS (P = 0.2546, HR = 1.2955, 95% CI, 0.8500-1.9746) were not significantly different (**Figures 6D–F**). When the patients were classified as those on no adjuvant chemotherapy, 3-month chemotherapy, and 6-month chemotherapy, irrespective of the levels of early postoperative CEA, the differences in OS, DFS, and CSS were not significant (all P-values were >0.05) (**Figures 7A–F**).

## Discussion

Patients with stage II CRC can be classified into IIA, IIB, and IIC according to the T stage, and the 5-year OS of stage II CRC can be about 80%. However, there was a significant difference in the prognosis between stages IIB/IIC and stage IIA, with 5-year OS of 72.2% and 83.4%, respectively (15). A study found that the recurrence risk in stage II CRC T4 patients is about twice that in T3 patients (16). Even the 5-year OS (71.5%) and DFS (70.0%) of IIB/IIC (T4N0) patients were lower than those of stage IIIA (T1-2N1) patients (87.7% and 84.0%, respectively) (17, 18). Therefore, the T stage is considered the most important prognostic factor in stage II CRC. Preoperative CEA was recognized as an independent prognostic factor for CRC (19, 20), while postoperative CEA was more important in the monitoring of recurrence and metastasis, and the postoperative CEA levels increased 2-6 months before the diagnosis of recurrence in 18%-75% of the recurrent CRC cases (10, 21, 22). It was more typical in intermediate diseases (stage II and III) (9, 10). In previous research, we found that postoperative CEA was an important prognostic factor for colon cancer that significantly improved the performance of the TNM model. The model combined postoperative CEA with T and N stages (TN-CEA) and was the best prognostic model for stage III colon cancer (11). Other studies have also supported the importance of postoperative CEA in tumor prognosis (9, 10, 12). Lin et al. suggested that patients with elevated postoperative CEA showed recurrence earlier. Park et al. suggested that preoperative and postoperative CEA were independent prognostic factors for tumor recurrence, and Wang et al. also suggested that postoperative CEA was an independent prognostic factor for DFS and OS in CRC, and a positive or elevated postoperative CEA indicated poor prognosis (9, 10, 12). However, these studies did not separately analyze patients with stage II CRC. The application value of postoperative CEA in stage II CRC is extremely important but it has not received enough attention. And considering the heterogeneity in the prognosis of stage II CRC, we thought it was necessary to conduct a separate study on the effects of postoperative CEA on the prognosis of stage II CRC.

We performed a single-center retrospective clinical analysis and found that elevated early postoperative CEA was the strongest independent prognostic factor for stage II CRC and was more significant than T stage and preoperative CEA. Patients with elevated early postoperative CEA had a worse prognosis. When we combined preoperative CEA and early postoperative CEA to get CEA ratio and CEA absolute value, the patients with CEA ratio ≤0.55 or CEA absolute value ≤-0.98 also showed a worse prognosis. The above results confirmed the prognostic guiding value of early postoperative CEA in stage II CRC, which is of great significance for the application of early postoperative CEA and the prognostic evaluation of stage 2 CRC. To the best of our knowledge, the current study is the first to evaluate the application value of early postoperative CEA in stage II CRC, which is important to guide prognosis evaluation and monitor stage II CRC patients.

It is worth noting that in this study, we set the CEA test time as before surgery and within 3 months after the surgery because of the half-life of CEA in the blood and the time whether to continue to receive adjuvant chemotherapy. The decision was also supported by another study (9). Patients who showed metastasis and recurrence during this period were excluded because these patients were considered to have synchronous metastases (23, 24).

There is no standard cutoff value of CEA at present and some reports suggested that 5 ng/ml was not the best cutoff value. Emile Tan et al. conducted a quantitative meta-analysis of 20 studies and found that when the CEA cutoff value was 2.2 ng/ml, it provided the best sensitivity and specificity for the monitoring of postoperative recurrence or metastasis (25). When using CSS as the endpoint to perform ROC, the early postoperative CEA cutoff value was 3.66 ng/ml and it predicted the prognosis of stage II CRC patients more accurately.

To remove the effect of other factors, we used PS-adjusted regression to adjust for the other factors, and the results were verified using PS stratification and IPTW. A previous study has shown that PS-adjusted regression is the most stable and accurate stratification method. This method is easy to use and can integrate multiple confounding factors through the PS score to reduce the interference of the confounding factors and ensure the accuracy of the results (26).

In previous studies, T4 stage and preoperative CEA were considered to have an important influence on the prognosis of stage II CRC (15, 19, 20, 27). Babcock et al. found that the T4 stage had the most negative impact on the survival of stage II colon cancer patients, and it was the most weighted HRF. When a single HRF was considered, only patients in stage T4 benefited from adjuvant chemotherapy, and patients with multiple HRFs benefited from adjuvant chemotherapy only when the HRFs included T4 (27). Researchers also proposed that the preoperative CEA and TNM stage had equal value and suggested that preoperative CEA should be included in the TNM stage to assess the prognosis of patients (21, 28–30). However, these views did not take into account postoperative CEA. Our study demonstrated that early postoperative CEA predicted and influenced the prognosis of stage II CRC stronger than T stage and preoperative CEA. This indicated that the value of early postoperative CEA is similar to T stage and preoperative CEA in the prognosis assessment and monitoring of stage II CRC and is worthy of further investigation. It also suggested that we should pay more attention to the changes in early postoperative CEA, which can provide a better idea for personalized diagnosis and treatment of the stage II CRC patients.

The role of adjuvant chemotherapy in stage II CRC patients has been the focus of researchers worldwide. A few previous studies have failed to evaluate significant survival benefits with respect to adjuvant chemotherapy in stage II CRC (31–33); however, others have suggested certain benefits of adjuvant chemotherapy for stage II CRC patients with HRFs (2, 3). At present, NCCN guidelines recommend adjuvant chemotherapy for stage II CRC patients with HRFs (6); however, postoperative CEA has not been included as an HRF. The guidelines recommend 6-month adjuvant chemotherapy for stage II CRC patients with HRFs (34, 35). Based on the results of the IDEA and TOSCA studies, 3-month adjuvant chemotherapy with CAPEOX may be considered for stage II CRC patients with HRFs except for T4 and low-risk stage III patients with T1-3N1 (36, 37). We applied early postoperative CEA to stratified patients. The results suggested that in patients which early postoperative CEA >3.66 ng/ml, compared to patients without adjuvant chemotherapy, patients with adjuvant chemotherapy showed an improved CSS (P = 0.040; HR = 0.6501; 95% CI, 0.4339 - 0.9741) after adjuvant chemotherapy. When the patients were classified based on the chemotherapy regimens, irrespective of the early postoperative CEA levels, the differences in OS, DFS, and CSS were not significantly different. Our results showed that there was insufficient evidence to support the suggestion that early postoperative CEA can be applied to the guidance of adjuvant chemotherapy for stage II CRC patients. The main reasons behind this conclusion were as follows: Firstly, the number of cases we considered was insufficient. Secondly, there were fewer deaths in the cases we considered. Finally, from 2007 to 2015, the inclusion criteria and chemotherapy regimens for adjuvant chemotherapy at our hospital lacked standardization. It is worth emphasizing that during the stratification of patients taking adjuvant chemotherapy for stage II CRC, early postoperative CEA showed possible trends and potential values, which are worthy of further investigation in a larger sample size.

There are certain shortcomings of this study. Firstly, we performed a retrospective study, and although it highlighted the importance of early postoperative CEA for patients with stage II CRC, the results may be less significant than a prospective study. Secondly, while considering the prognostic factors for stage II CRC, we did not consider the microsatellite stability, and MSI-H/DMMR is currently recognized as a low-risk factor for stage II CRC (38). Thirdly, other studies suggested that the number of tumor markers that increase after surgery had an impact on the prognosis of patients (39) but we considered CEA only. Fourthly, this is a single-center study, and data from other centers were not used for verification. Lastly, we did not make a clear distinction between the colon and rectal cancer.

## Conclusion

Early postoperative CEA was a better biomarker for prognosis of stage II CRC patients than T stage and preoperative CEA, and has the potential to become a high-risk factor to guide the prognosis and treatment of stage II CRC patients.

## Data Availability Statement

The raw data supporting the conclusions of this article will be made available by the authors, without undue reservation.

## Ethics Statement

The studies involving human participants were reviewed and approved by the ethics committee of the Harbin Medical University Cancer Hospital. The patients/participants provided their written informed consent to participate in this study.

## Author Contributions

LYL and DF had full access to all the data and take responsibility for the integrity of the data and accuracy of the data analysis. Conceptualization, LYL and DF. Data curation, DF, LYP, ZQ, YC, SW, XH, XT, GJ, XW, BX and JC. Formal analysis, LYP, ZQ, JX, BJ, XW, LY, and BX. Investigation, DF, YC, SW, XT, and GJ. Supervision, LYL. Writing, original draft, DF. Review and editing, DF, and LYL. All authors contributed to the article and approved the submitted version.

## Funding

The current work was supported by the Harbin Medical University Cancer Hospital Preeminence Youth Fund (no. JCQN2019-04) and the Medical Wisdom Research Fund by the Heilongjiang Sunshine Health Foundation (H21L0802).

## Conflict of Interest

The authors declare that the research was conducted in the absence of any commercial or financial relationships that could be construed as a potential conflict of interest.

## Publisher’s Note

All claims expressed in this article are solely those of the authors and do not necessarily represent those of their affiliated organizations, or those of the publisher, the editors and the reviewers. Any product that may be evaluated in this article, or claim that may be made by its manufacturer, is not guaranteed or endorsed by the publisher.

## Supplementary Material

Supplementary Figure 1 Overall survival (OS), disease-free survival time (DFS), and cancer-specific survival (CSS) based on the combination of preoperative CEA and early postoperative CEA.

Supplementary Figure 2 The receiver operating characteristics (ROC) curves of the CEA ratio and CEA absolute value with respect to OS.

Supplementary Figure 3 Overall survival (OS), disease-free survival time (DFS), and cancer-specific survival (CSS) based on the CEA ratio and CEA absolute value.
