# Supplementary figures and images for: Early Postoperative Serum Carcinoembryonic Antigen Is a Stronger Independent Prognostic Factor for Stage II Colorectal Cancer Patients Than T4 Stage and Preoperative CEA
Source: Front Oncol. 2022 Jan 11;11:758509. doi: 10.3389/fonc.2021.758509 (PMC8786716; doi:10.3389/fonc.2021.758509)

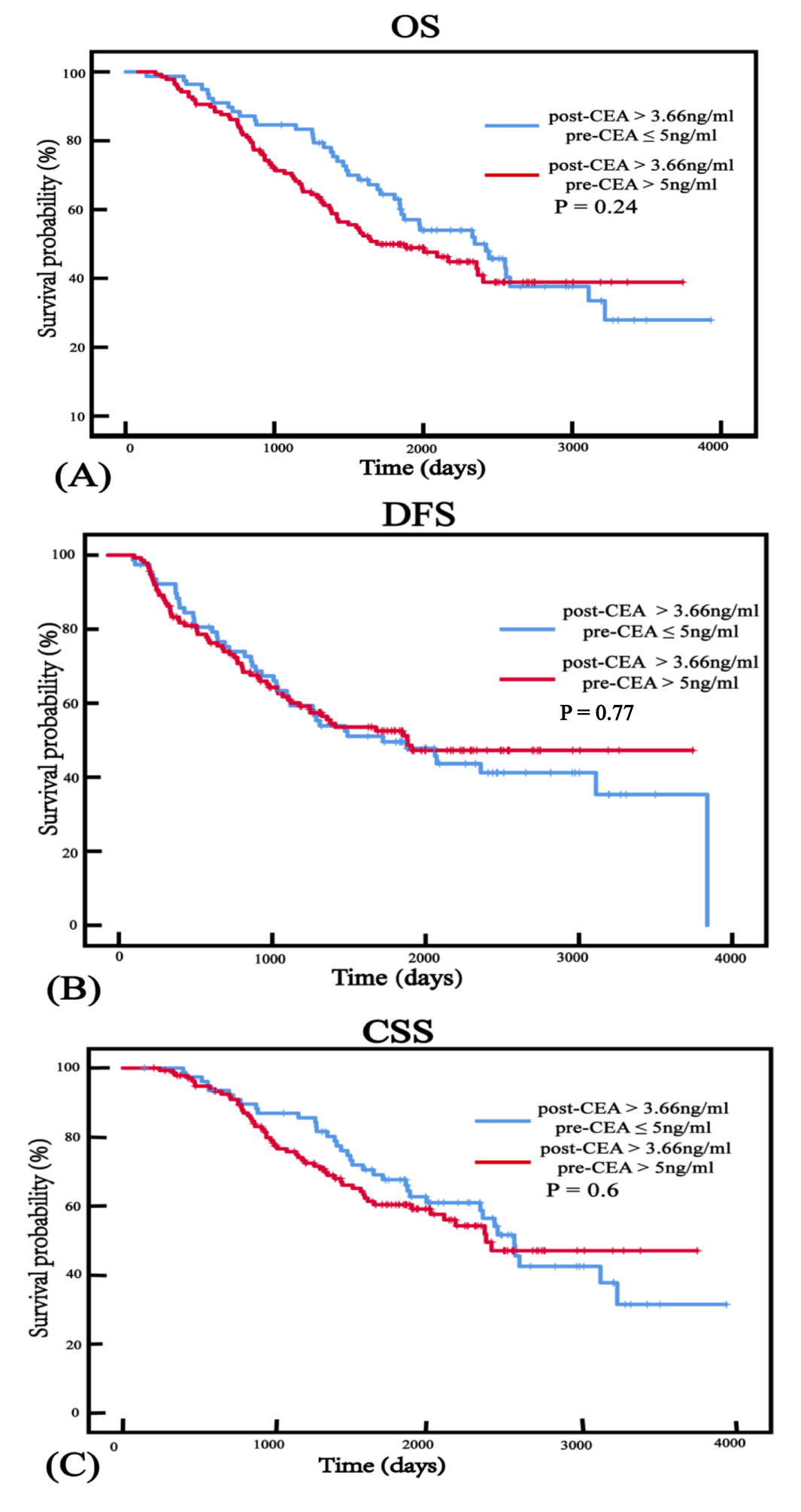

Supplement: Supplementary Figure 1 — Overall survival (OS), disease-free survival time (DFS), and cancer-specific survival (CSS) based on the combination of preoperative CEA and early postoperative CEA. [file Image_1.tif]

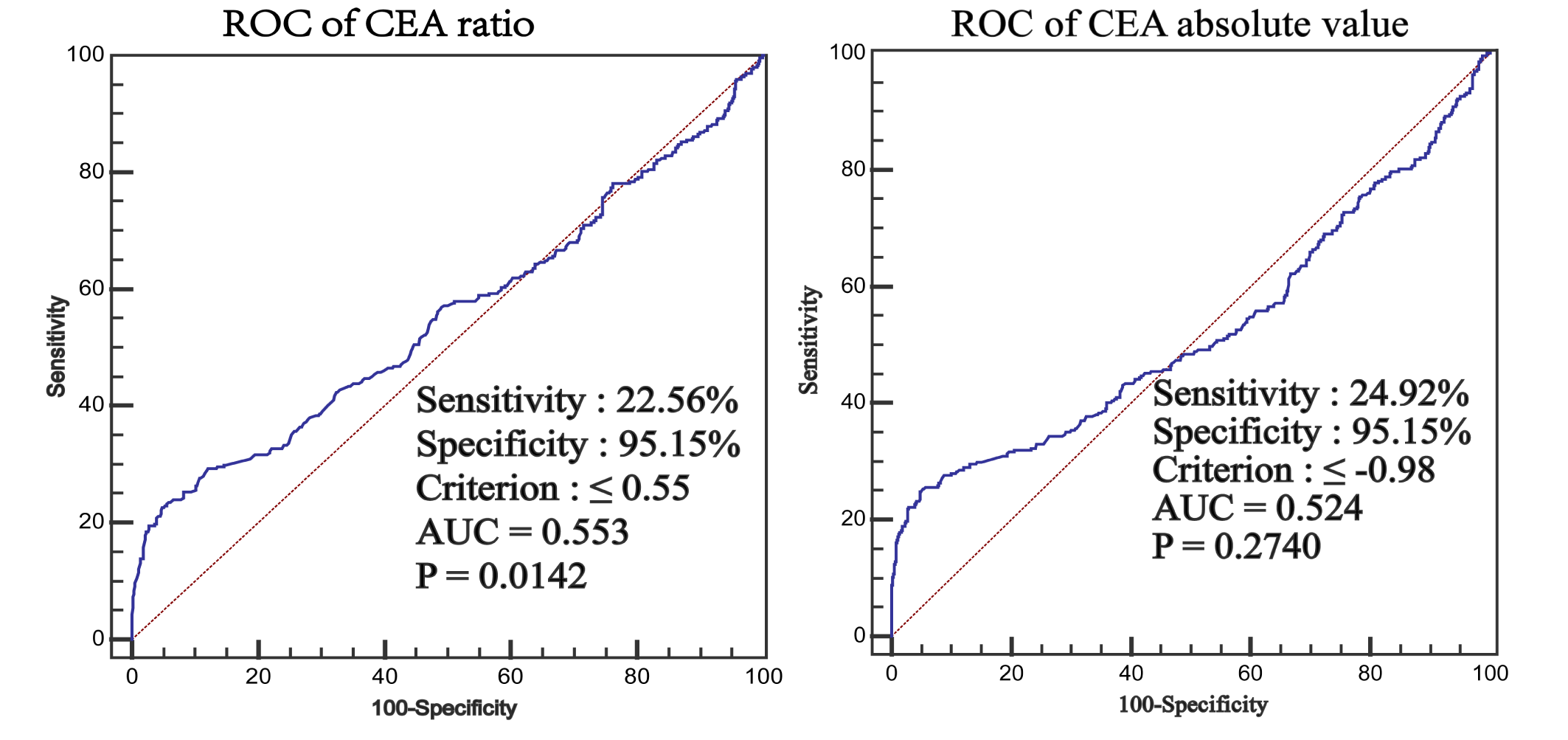

Supplement: Supplementary Figure 2 — The receiver operating characteristics (ROC) curves of the CEA ratio and CEA absolute value with respect to OS. [file Image_2.tif]

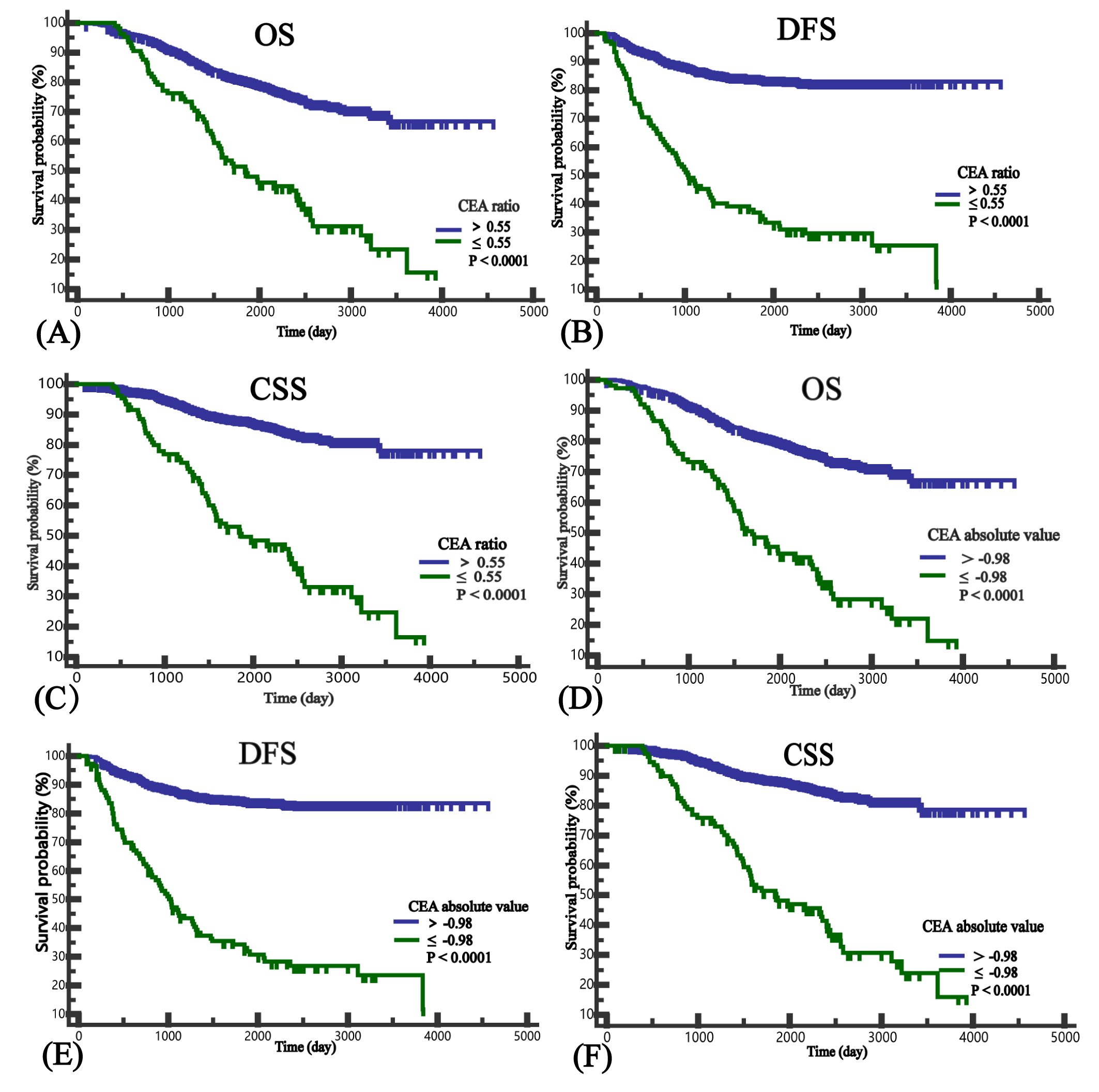

Supplement: Supplementary Figure 3 — Overall survival (OS), disease-free survival time (DFS), and cancer-specific survival (CSS) based on the CEA ratio and CEA absolute value. [file Image_3.tif]
